# The Utility of Small Fishes for the Genetic Study of Human Age-Related Disorders

**DOI:** 10.3389/fgene.2022.928597

**Published:** 2022-07-15

**Authors:** Eisuke Dohi, Hideaki Matsui

**Affiliations:** Department of Neuroscience of Disease, Brain Research Institute, Niigata University, Niigata, Japan

**Keywords:** small fishes, zebrafish, medaka, turquoise killifish, age-related disorders, genomes

## Abstract

Animal models have been used to model human diseases, and among them, small fishes have been highlighted for their usefulness in various ways, such as the low cost of maintenance, ease of genetic modification, small size for easy handling, and strength in imaging studies due to their relative transparency. Recently, the use of turquoise killifish, *Nothobranchius furzeri*, which is known to exhibit various aging phenotypes in a short period, has attracted attention in research on aging and age-related diseases. However, when using animal models, it is important to keep their genetic background and interspecies differences in mind for translating them into human diseases. In this article, we obtained the gene symbols of protein-coding genes of turquoise killifish, medaka, zebrafish, and humans from NCBI datasets and extracted common shared genes among four species to explore the potential of interspecies translational research and to apply small fish models for human age-related disorders. Common shared protein-coding genes were analyzed with the Reactome Pathway Database to determine the coverage of these genes in each pathway in humans. We applied common shared genes to the Orphanet database to establish a list of human diseases that contain common shared genes among the four species. As examples, the senescence-related pathways and some pathways of human age-related diseases, such as Alzheimer’s disease, Parkinson’s disease, frontotemporal dementia, nonalcoholic fatty liver disease, progeria, hepatocellular carcinoma, and renal cell carcinoma, were extracted from the curated pathway and disease list to discuss the further utility of fish models for human age-related disorders.

## Introduction

Zebrafish produce more than 100 eggs per oviposition, and medaka and turquoise killifish, *Nothobranchius furzeri*, also produce many but fewer eggs than zebrafish (Furutani-Seiki and Wittbrodt, 2004; Skinner and Watt, 2007; Polacik et al., 2016). Because small fish have a larger number of fertilized eggs than mice and because spawning and fertilization take place outside the parent’s body, it is very easy to microinject the desired gene-editing factor into the egg. The recent emergence of the CRISPR–Cas9 technique (Hwang et al., 2013; Ablain et al., 2015; Varshney et al., 2015) allows us to quickly knock out or edit specific genes in various organisms compared with zinc-finger nucleases (ZFNs) (Doyon et al., 2008; Meng et al., 2008) or transcription activator-like effectors (TALENs) (Huang et al., 2011; Sander et al., 2011). The CRISPR–Cas9 system also enables the knock-in of a specific DNA sequence mediated by homology-directed repair (HDR) or other mechanisms (Irion et al., 2014; Prykhozhij et al., 2018). These gene-editing techniques are applicable for zebrafish, medaka, and turquoise killifish. Because of the relatively hard chorion of turquoise killifish compared with zebrafish and medaka, it might be difficult to perform microinjection into the eggs of turquoise killifish. However, the methods of microinjection into the egg of turquoise killifish are being improved (Valenzano et al., 2011; Hartmann and Englert, 2012), and the use of genetic engineering in turquoise killifish has been expanding (Allard et al., 2013; Harel et al., 2016).

Turquoise killifish is a small fish species native to Africa, primarily Mozambique, that lives in ponds, swamps, and puddles (Poeschla and Valenzano, 2020). There is a short rainy season and a long dry season when adult fish cannot survive because the water dries up. Although adult fish cannot survive, turquoise killifish survive as a species through drought-resistant eggs laid in the soil, which can hatch during the future rainy season. In such a life cycle, turquoise killifish may not be subjected to a positive selection pressure to acquire various antiaging properties (Cui et al., 2019). Therefore, the lifespan of turquoise killifish is approximately four to six months, which is very short compared to zebrafish and medaka (Polacik et al., 2016). Around the age of three months, the turquoise killifish exhibits several signs of aging, such as organ atrophy, spine curvature, and increased levels of senescence-associated beta-galactosidase (Genade et al., 2005; Valenzano et al., 2006; Harel et al., 2015). Given their aging phenotypes, we examined the central nervous system of turquoise killifish and found that this fish showed age-dependent degeneration of dopaminergic and noradrenergic neurons, with gradually progressing alpha-synuclein pathology (Matsui et al., 2019). These pathological findings are similar to those of human Parkinson’s disease, and very interestingly, genetic depletion of alpha-synuclein with the CRISPR–Cas9 system mitigates neurodegeneration (Matsui et al., 2019). These findings suggest that alpha-synuclein can be a causative protein in the pathogenesis of Parkinson’s disease, and turquoise killifish could be a useful tool for unveiling the mechanisms of Parkinson’s disease and hopefully other age-related diseases.

To further utilize the potential of translational research of such fish models, it is important to know the genetic background of each small fish compared to those of humans and other small fishes. In this article, we analyzed the genetic backgrounds of turquoise killifish, medaka, zebrafish, and humans (Reichard et al., 2009; Kirchmaier et al., 2015; Valenzano et al., 2015; Delomas and Dabrowski, 2018; Poeschla and Valenzano, 2020) and explored the utility of small fish for translational research of human age-related disorders.

## Methods

### Finding the Common Shared Genes

Gene symbols of each species were extracted from protein-coding genes in the NCBI datasets (https://www.ncbi.nlm.nih.gov/datasets/; accessed on 18th January). The gene symbols were capitalized as normalization to extract the common shared genes by generating a Venn diagram in the exact match manner (https://bioinformatics.psb.ugent.be/webtools/Venn/).

Given another round of whole-genome duplication in teleosts, some genes did not correspond between humans and fishes in a 1:1 manner, and two orthologues could be present in the teleost fishes. To extract such duplicated genes in fishes, we first extracted fish genes that did not overlap with human genes in an exact match manner. Then, the gene symbols ending with A or B were picked up. After depleting the last letter, the genes with equal combinations of the remaining strings were considered to be a pair of duplicated genes. These extracted duplicated genes were examined to determine whether orthologues overlapped with human genes. This procedure was repeated through four species to find additional common shared genes.

### Coverage of Common Shared Genes in Each Human Pathway

Common shared genes were applied to the Reactome pathway database (Jassal et al., 2020) to determine the coverage of genes in each human pathway.

### Extraction of Orphanet Codes That Contain Common Shared Genes

Metascape (Zhou et al., 2019) was utilized by applying the Orphanet database (https://www.orpha.net/consor/cgi-bin/index.php). The list of ORPHAcodes related to each common shared gene was obtained, and the list was organized according to the list with the index of each ORPHAcode.

## Results

### Common Shared Genes Among Humans and three Fishes

Approximately 500 million years ago, vertebrates, including humans, experienced a whole-genome duplication in which the genome doubled twice in our ancestors (Ohno, 1970; Dehal and Boore, 2005). Another round of whole-genome duplication occurred in teleosts, including zebrafish, medaka, and turquoise killifish (Chiu et al., 2004; Hoegg et al., 2004; Jaillon et al., 2004). This is one of the most significant genetic differences between humans and fishes. Among these three fishes, sex chromosomes have not been identified in zebrafish. Zebrafish sex determinants remain unclear, but environmental factors are known to affect zebrafish sex determination (Baroiller and D'Cotta, 2001; Orn et al., 2003; Abozaid et al., 2012). Similar to humans, medaka or turquoise killifish sex is determined by XX/XY sex chromosomes (Schartl, 2004; Valenzano et al., 2009; Reichwald et al., 2015), which is another major genomic difference among the four species.

**FIGURE 1:**
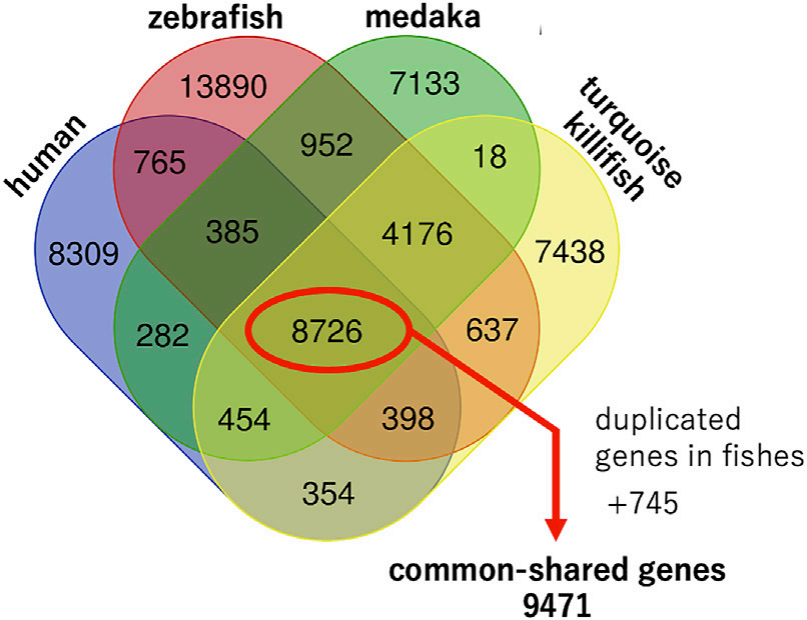
Genomic characteristics of humans, zebrafish, medaka, and turquoise killifish. Venn diagram generated by an exact match with gene symbols normalized as strings. In addition to 8,726 common shared genes, duplicated orthologues in fishes were extracted and an additional 745 common shared genes were found. In total, 9,471 common shared genes were found among the four species.

We attempted to extract the gene symbols of each species from public databases, such as the UniProt (UniProt, 2021), Ensembl genome browser (Howe et al., 2021), and NCBI datasets, and found that the NCBI datasets contained the most gene symbols of each species. Therefore, we extracted the gene symbols of each species from the NCBI datasets (https://www.ncbi.nlm.nih.gov/datasets/; accessed on 18 January) based on the protein-coding genes. The numbers of protein-coding genes were 19,671; 29,961; 22,140; and 22,207 for human, zebrafish, medaka, and turquoise killifish, respectively (Table 1; Supplementary Table S1). A Venn diagram was generated by exact matching with gene symbols normalized as strings (https://bioinformatics.psb.ugent.be/webtools/Venn/), and 8,726 genes were found to be common among the four species (Figure 1).

**TABLE 1:** Lifespans and genomic characteristics of humans, zebrafish, medaka, and turquoise killifish.

	Human	Zebrafish	Medaka	Turquoise killifish
Scientific name	*Homo sapiens*	*Dania rerio*	*Oryzias latipes*	*Nothobranchius furzeri*
Life span	100 years	2–5 years	2–5 years	4–6 months
Genome size	3100 Mbp	1373 Mbp	734 Mbp	1242 Mbp
Number of chromosomes	23 chromosomes	25 chromosomes	23–24 chromosomes	19 chromosomes
	(2n = 46)	(2n-50)	(2n-46–48)	(2n-38)
Sex determination	XX/XY	Environmental?	XX/XY	XX/XY
**Reference genome**	**GRCh38.p13**	**GCRz11**	**ASM223467v1**	**Nfu_20140520**
Protein-coding genes	19,671	29,961	22,140	22,207
Small RNAs	1,227	3,899	1,223	89
Pseudogenes	16,570	329	188	324
Noncoding	17,654	9,205	3,270	2,515
Other	8,028	304	105	42

The genomic characteristics of four species were extracted from the NCBI genome datasets (https://www.ncbi.nlm.nih.gov/datasets/; accessed on 18th January).

As mentioned previously, teleost fishes experienced an additional round of whole-genome duplication compared to humans, and so, some genes did not correspond one-to-one between humans and fish. In such cases, two orthologues could be present in the teleost fishes. We extracted such duplicated orthologues from fishes and found an additional 745 common shared genes among four species out of a total of 9,471 genes (Supplementary Table S2). A study comparing short- and long-lived turquoise killifish strains showed that long-lived killifish acquired specific SNPs in several aging genes (*grn*, *tnfb*, *pdgfr*, *brca1*, *tp53*, *bp*, *ercc6*, *ghr*, *irs4*, *foxo4*, *myc*, *egr1*, *med1*, *ncor1*, *polg*, *gsr*, *mgat5*, *tert*, *hsf1*, and *hspa9*) under positive selection (Valenzano et al., 2015). Among these 19 genes, 16 genes were included in the common shared genes except for *brca1*, *irs4*, and *tnfb*.

### Human Pathways and Diseases With Common-Shared Genes

Then, the 9,471 common shared genes were applied to the Reactome Pathway Database (Jassal et al., 2020), a web tool for human pathway analysis, to determine the proportion and number of common shared genes in known human biological pathways. A total of 2,405 pathways were found to be associated with at least one of the common shared genes. Given the list of 2,405 pathways, we extracted some of the senescence-related pathways and noticed that common shared genes covered approximately 30–50% of the genes in the human senescence-related pathways (Table 2). Complete gene lists in each pathway are available in Supplementary Table S3. Next, we applied the 9,471 common shared genes to the Orphanet database (https://www.orpha.net/consor/cgi-bin/index.php) (Weinreich et al., 2008) to establish the human disease list, which included at least one gene from the common shared genes. From this application, 2,677 Orphacodes were extracted. We extracted several human age-related disorders, such as Alzheimer’s disease, Parkinson’s disease, frontotemporal dementia, nonalcoholic fatty liver disease, and progeria, from our Orphanet disease list containing common shared genes. We also extracted hepatocellular carcinoma and renal cell carcinoma because turquoise killifish are known to develop hepatocellular carcinoma and renal cell carcinoma at high rates despite their short lifespan (Di Cicco et al., 2011) (Table 3; Supplementary Table S4).

**TABLE 2:** Senescence pathway from the common shared genes.

Pathways identifier	Senescence-related pathways	#Entities found	#Entities total	#Interactors found	#Interactors total
R-MSA-2559583	Cellular senescence	87	200	335	662
R-HSA-2559580	Oxidative stress–induced senescence	40	114	178	357
R-HSA-2559582	Senescence-associated secretory phenotype (SASP)	39	91	23	48
R-H5A-2559586	DNA damage/telomere stress-induced senescence	19	71	135	248
R-H SA-2558585	Oncogene-induced senescence	23	42	79	154
R-HSA-2559584	Formation of senescence-associated heterochromatic foci (SAHF)	5	17	59	116
R-HSA-9630747	Disease of cellular senescence	2	4	22	38

The pathway searched with “senescence.” The search word “senescence” was applied to the pathway list of common shared genes (Supplementary Table S3), and part of the results is presented as an example.

**TABLE 3:** List of genes related to some age-related disorders.

ORPHAcode	Search with “Alzheimer”	Genes in common shared genes (not in common shared genes)
ORPHA:1020	Early-onset autosomal dominant Alzheimer's disease	*TOMM40*, *ABCA7*, *PSEN2*, *SOL1*, *PSEN1*, *(APP*, *TREM2)*
ORPHA:238616	Non-rare in Europe: Alzheimer's disease	*ABCA7*, *SORL1*, *APOE*, *(TREM2)*
**ORPHAcode**	**Search with “Parkinson”**	**Genes in common shared genes (not in common shared genes)**
ORPHA:2,828	Young-onset Parkinson disease	*DNAJC6*, *PARK7*, *LRRK2*, *UCHL1*, *PODX1*, *SYNJ1*, *VSP13C*, *PINK1*, *PRKN*, *(HTRA2*, *SNCA)*
ORPHA:53351	X-linked dystonia-Parkinsonism	*TAF1*
ORPHA:71517	Rapid-onset dystonia-Parkinsonism	*ATP1A3*
ORPHA:90020	Parkinson–dementia complex of Guam	*PARK7*, *TPRM7*
ORPHA:98933	Multiple system atrophy Parkinsonian type	*COQ2*
ORPHA:171695	Parkinsonian-pyramidal syndrome	*FBXO7*, *(SNCA)*
ORPHA:199351	Adult-onset dystonia-Parkinsonism	*PLA2G6*
ORPHA:238455	Infantile dystonia-parkinsonism	*SLC6A3*
ORPHA:319705	Non-rare in Europe: Parkinson’s disease	*GBA*
ORPHA:363654	X-linked parkinsonism-spasticity syndrome	*ATP6P2*
ORPHA:391411	Atypical juvenile parkinsonism	*DNAJC6*, *PODXL*, *SYNJ1*
ORPHA:411602	Hereditary late-onset Parkinson disease	*LRK2*, *GIGYF2*, *GBA*, (*DNAJC13*, *EIF4G1*, *SNCA*, *VPS3S*)
ORPHA:521406	Dystonia-Parkinsonism-hypermanganesemia syndrome	*SLC39A14*
**ORPHAcode**	**Search with “frontotemporal dementia”**	**Genes in common shared genes (not in common shared genes)**
ORPHA:52430	Inclusion of body myopathy with Paget disease of bone and frontotemcioral dementia	*VCP*, *(HNRNPA1*, *HNROA2B1)*
ORPHA:275864	A behavioral variant of frontotemporal dementia	*VCP*, *SQSTM1*, *GRN*, *PSEN1*, *(O9ORF72*, *CHMP2B*, *MAPT*, *TMEM106B*, *TPEM2)*
ORPHA:275872	Frontotemporal dementia with motor neuron disease	*VCP*, *SQSTM1*, *TBK1*, *TARDBP (O9ORF72*, *CHCD10*, *FUS)*
**ORPHAcode**	**Search with “Non-alcoholic fatty liver disease”**	**Genes in common-shared genes (not in common-shared genes)**
ORPHA:33271	Non-rare in Europe: Non-alcoholic fatty liver disease	*PNPLA3*, (*APOC3*)
**ORPHAcode**	**Search with “Progeria”**	**Genes in common shared genes (not in common shared genes)**
ORPHA:740	Hutchinson–Gilford progeria syndrome	*LMNA*, *ZMPSTE24*
ORPHA:280576	Nestor–Guillermo progeria syndrome	*BANF1*
ORPHA:363618	LMNA-related cardiocutaneous progeria syndrome	*LMNA*
**ORPHAcode**	**Search with “hepatocellular carcinoma”**	**Genes in common shared genes (not in common shared genes)**
ORPHA:33402	Pediatric hepatocellular carcinoma	*CNNTB1*, *MET*
ORPHA:210159	Adult hepatocellular carcinoma	*PIK3CA*, *PDGFRL*, *AXIN1*, *CTNNB1*, *EGF*, *TSC2*, *CASP8*, *TP53*, (*TSC1*)
ORPHA:435953	Proseroid features—hepatocellular carcinoma predisposition syndrome	*SPRTN*
**ORPHAcode**	**Search with “renal cell carcinoma”**	**Genes in common shared genes (not in common shared genes)**
ORPHA:47044	Hereditary papillary renal cell carcinoma	*MET*
ORPHA:319294	Papillary renal cell carcinoma	*MET*, *(MITF)*
ORPHA:319393	Chronophage renal cell carcinoma	*HNF1A*, *(MITF*, *PBRW1*, *TMEM127)*
ORPHA:319308	MiT family translocation renal cell carcinoma	*ASPSCRl*, *TFE3*, *SFPQ*, *TFEB*, *PRCC*, *(CLTC*, *NONO*)
ORPHA:404511	Clear-cell papillary renal cell carcinoma	*HNF1F*, *PBRM1*, (*MITF*, *TMEM127*)
ORPHA:422526	Hereditary clear-cell renal cell carcinoma	*OGG1*, *SLC49A4*, *RNF139*, *HSPBAP1*, *FLCN*, (*DIR3*, *FHIT*)

The list of age-related disorders from Orphanet according to common shared genes. The search words “Alzheimer,” “Parkinson,” “frontotemporal dementia,” “nonalcoholic,” “progeria,” “hepatocellular carcinoma,” and “renal cell carcinoma” were applied to the list of Orphanet codes, which included at least one common shared gene (Supplementary Table S4). Search results are presented as age-related disorders. Gene symbols enclosed in parentheses () are disease-related genes that were not found in the common shared gene.

Based on these lists obtained previously, several age-related disease genes were explored. *APOE* is known to be associated with Alzheimer’s disease and is located in the lifespan-related loci of the turquoise killifish (Kirschner et al., 2012). *APOE* was also reported as an aging marker of the short-lived fish *N. guentheri* (Wang et al., 2014). *PARK7* is one of the causative genes of familial Parkinsonism, and polymorphisms in *PARK7* were reported to be related to the lifespan of turquoise killifish (Genade and Wilcox, 2021). *GRN*, one of the causative genes of frontotemporal dementia, is known to be regulated downstream of *PARK7* in the context of neuroprotection (Genade and Wilcox, 2021). A common biological pathway linking *PINK–PARKIN–PARK7* was reported in the pathogenesis of Parkinson’s disease (van der Merwe et al., 2015), and these three genes were observed among the common shared genes. A search on Orphanet for “Parkinson” seems to cover the common shared genes well, except for hereditary late-onset Parkinson’s disease (ORPHA:411602). Referring to the gene list of turquoise killifish, *dnaj13*, *eif4g1*, and *vsp35* were found, but the orthologous genes corresponding to human *SNCA* and human *HTRA2* were not found. Although *snca* was not included in the gene list of turquoise killifish constructed with NCBI datasets, *snca* is known to be expressed in turquoise killifish (Matsui et al., 2019) and was found in turquoise killifish in the UniProt database (UniProt, 2021). Thus, turquoise killifish possesses most of the Parkinson’s disease-related genes, which could be applied to translational research on Parkinson’s disease. Compared to age-related diseases, common shared genes only covered 30–50% of the genes in senescence-related pathways. This difference in gene coverage may be due to the relatively small number of genes in common or to genomic differences that occur during evolution. In interpreting the effects of aging on the pathogenesis of age-related diseases using fish as a model, it will be necessary to keep in mind that the effects may be species dependent.

## Discussion

In this article, we explored the genetic background and common shared genes among humans and three fishes by using the available gene symbol data from the NCBI database (https://www.ncbi.nlm.nih.gov/datasets/). We applied common shared genes to the Reactome pathway database (Jassal et al., 2020) to determine the coverage of genes in human pathways. To determine the involvement of commonly shared genes in human diseases, we utilized the Orphanet database (Weinreich et al., 2008). These genetic background data between humans and fishes are important and worth considering before translating the previous findings of fish models in the context of human disease studies, including aging studies. Additionally, these data allow us to design an experiment that applies a fish model including turquoise killifish in aging studies. Although this kind of knowledge-based approach allows us to observe the genomic landscape from a broad perspective in an interspecies manner, several caveats and limitations exist.

First, in the available database, even among protein-coding genes, fairly many genes are still waiting to be annotated in many species. As observed in turquoise killifish, *snca* was not obtained from NCBI datasets. A blastp search (Altschul et al., 1990; States and Gish, 1994; Boratyn et al., 2012) of the amino acid sequence of human *SNCA* based on the turquoise killifish protein dataset yielded the answer “PREDICTED: alpha-synuclein-like” (Supplementary Table S5). This might be the reason why the *Snca* protein of turquoise killifish has not been annotated and could not be found in the list of gene symbols in the NCBI dataset. There are many gene symbols beginning with LOC, a notation that indicates that a published symbol is not available for this gene, and orthologues have not yet been determined (https://www.ncbi.nlm.nih.gov/books/NBK3840/#genefaq.Conventions). The numbers of gene symbols beginning with LOC are 266; 4,473; 6,924; and 7,332 for human, zebrafish, medaka, and turquoise killifish, respectively. This caveat of a lack of annotation should be kept in mind when attempting to explore genomic information.

Second, pseudogenes, miRNAs, noncoding RNAs, and others would be worth investigating (Esteller, 2011; Cheetham et al., 2020; Statello et al., 2021); we did not explore such RNAs in an interspecies manner in this study. It is often difficult to obtain sufficient insight by comparing such genes in an interspecies manner. Thus, some meaningful information may be overlooked.

Third, whole-genome alignment is suitable for covering the entire genomes of multiple species. However, whole-genome comparison studies among multiple species require long computation times, complex algorithms, and expensive computational resources and are difficult for researchers not familiar with bioinformatics to repeat in a timely manner (Armstrong et al., 2019). If the target gene has already been determined, blastp (Altschul et al., 1990; States and Gish, 1994; Boratyn et al., 2012) is an effective way to evaluate coverage, e-values, and percent identity by using human sequence data as a reference. It should be noted that a gene may have multiple amino acid sequences, and a single amino acid sequence may be annotated to multiple genes in the protein dataset of the reference organism. For example, medaka and turquoise killifish Tp53 amino acid sequences showed higher homology to human TP63 and human TP73 (TP53 family proteins) (Belyi et al., 2010) than to human TP53 (Supplementary Table S6).

Finally, in the disease-related genes, we only focused on Orphanet (Weinreich et al., 2008); genes potentially involved in disease modification were not included. Such regulatory genes would be better to be included when we consider applying an animal model for specific diseases. In addition, there are many variants of uncertain significance (VUS) (Elliott, 2020; Sullivan, 2021) in human genes, and their evaluation is also rapidly progressing (Mahecha et al., 2022; Postel et al., 2022). Thus, the identification of disease-related genes will be increasing, and their significance will be determined in the future.

Given the current situation, timely updates with recent genomics data would be ideal to translate and interpret the data and phenomena with model animals. A goal should be to establish a platform where anyone can easily compare genomes between species, even if they cannot write the code for analysis.

## Data Availability

Publicly available datasets were analyzed in this study. This data can be found here: https://www.ncbi.nlm.nih.gov/datasets/.
